# Metallosis after Hip Arthroplasty Damages Skeletal Muscle: A Case Report

**DOI:** 10.3390/geriatrics8050092

**Published:** 2023-09-15

**Authors:** Roberto Bonanni, Lorenzo Abbondante, Ida Cariati, Elena Gasbarra, Umberto Tarantino

**Affiliations:** 1 Department of Biomedicine and Prevention, “Tor Vergata” University of Rome, Via Montpellier 1, 00133 Rome, Italy; roberto.bonanni1288@gmail.com; 2 Department of Orthopaedics and Traumatology, “Policlinico Tor Vergata” Foundation, Viale Oxford 81, 00133 Rome, Italy; lorenzo.abbondante@gmail.com (L.A.); gasbarra@med.uniroma2.it (E.G.); umberto.tarantino@uniroma2.it (U.T.); 3 Department of Systems Medicine, “Tor Vergata” University of Rome, Via Montpellier 1, 00133 Rome, Italy; 4 Department of Clinical Sciences and Translational Medicine, “Tor Vergata” University of Rome, Via Montpellier 1, 00133 Rome, Italy; 5 Centre of Space Bio-Medicine, “Tor Vergata” University of Rome, Via Montpellier 1, 00133 Rome, Italy

**Keywords:** metallosis, hip arthroplasty, muscle damage, oxidative stress, sarcopenia, fat infiltration, heavy metals

## Abstract

Good musculoskeletal quality dramatically influences the outcome of an arthroplasty operation in geriatric patients, as well as is a key element for optimal osseointegration. In this context, metallosis is a complication associated with the type of prosthesis used, as implants with a chromium–cobalt interface are known to alter the bone microarchitecture and reduce the ratio of muscle to fat, resulting in lipid accumulation. Therefore, the aim of our study was to investigate possible muscle changes by histological, morphometric, and immunohistochemical analyses in a patient undergoing hip replacement revision with elevated blood and urinary concentrations of chromium and cobalt. Interestingly, the muscle tissue showed significant structural changes and a massive infiltration of adipose tissue between muscle fibers in association with an altered expression pattern of important biomarkers of musculoskeletal health and oxidative stress, such as myostatin and NADPH Oxidase 4. Overall, our results confirm the very serious impact of metallosis on musculoskeletal health, suggesting the need for further studies to adopt a diagnostic approach to identify the cause of metallosis early and eliminate it as part of the prosthesis revision surgery.

## 1. Introduction

In recent years, the impact of heavy metals on musculoskeletal health has been significantly investigated [1,2]. Several pieces of evidence have shown that the presence of heavy metals negatively influences bone quality by damaging the microarchitecture and structural integrity [3], whereas knowledge regarding the effect of heavy metals on muscle tissue is still rather limited. In this context, chronic cadmium exposure of C57BL/6J mice has been shown to significantly reduce the ratio of gastrocnemius muscle to body weight, dramatically increasing pro-inflammatory cytokine production and lipid accumulation [4]. This evidence has had a significant impact on the orthopedic field, influencing the choice of prostheses and synthetic means capable of preserving bone and muscle quality [5]. Indeed, good musculoskeletal quality promotes osseointegration and reduces the risk of complications, counteracting disability and mortality in geriatric patients undergoing orthopedic surgery [6,7]. In this regard, Ikeda et al. described the case of a 56-year-old woman undergoing hip arthroplasty with a cobalt–chromium alloy prosthesis who developed progressive sensory disturbances, hearing loss, and hypothyroidism caused by metallosis [8].

Although polyneuropathy from cobalt–chromium intoxication is one of the worst-case scenarios, chronic infiltration of metal debris can lead to chronic local inflammatory changes as well as broad-spectrum systemic clinical reactions [9]. In such a context, the musculoskeletal tissue may be drastically affected by an aberrant concentration of metal ions and experience muscle atrophy and increased fat infiltration [4]. The deterioration of skeletal muscle tissue caused by metallosis due to arthroplasty surgery may accelerate the muscle mass loss that is generally observed in geriatric patients [10,11]. Indeed, generalized loss of muscle mass, strength, and function, a condition described as sarcopenia, is one of the main complications encountered in elderly patients undergoing orthopedic surgery [12]. This age-related musculoskeletal pathology is characterized by altered expression patterns of multiple myokines that regulate and control muscle growth [13,14,15]. Of these, myostatin has certainly been indicated as being responsible for the increased atrophy of muscle fibers, as increased levels of this myokine have been detected in the muscles of elderly patients with sarcopenia [16]. Interestingly, a key role for myostatin has also been proposed in the crosstalk between skeletal muscle tissue and surrounding adipose tissue [17] since inhibition of myostatin signaling, specifically in skeletal muscle but not in adipose tissue of transgenic mice, was correlated with a reduction in fat mass and an increase in skeletal muscle mass [18]. In addition, myostatin has been suggested to promote adipogenesis and inhibit myogenesis in vitro by increasing cell cycle inhibitor activity [19].

Of note, sarcopenia also involves other cellular and molecular alterations, including increased oxidative stress resulting from the accumulation of reactive oxygen species (ROS), which exert negative effects on myogenic differentiation and survival, causing loss of muscle mass and function [20]. NADPH oxidase 4 (NOX4) is among the main enzymes responsible for ROS generation and appears to be highly expressed in skeletal muscle tissue, playing a primary role in myogenic differentiation and oxidative stress when overexpressed [21]. In this regard, Wu et al. simulated the sarcopenic condition in C2C12 murine muscle cells by treatment with benzo[a]pyrene, a toxic carcinogen, and found an increase in myostatin expression in association with increased production of reactive oxygen species (ROS) induced by NADPH Oxidase 2 (NOX2) and NADPH Oxidase 4 (NOX4) [22]. Interestingly, increased oxidative stress in muscle fibers could be the triggering event for other molecular alterations that characterize sarcopenia, such as mitochondrial dysfunction [23], nuclear apoptosis induction [24], as well as loss of the satellite cell pool and regenerative potential [25]. In elderly patients, all these conditions can promote a pro-inflammatory environment, worsening the outcomes of sarcopenia [26]. In fact, the cytokine storm triggered to promote muscle regeneration causes an increase in cytolytic and cytotoxic molecules and ROS in damaged muscle, aggravating tissue injury and damaging surrounding healthy tissue. Therefore, oxidative stress and inflammation are considered hallmarks of sarcopenia and represent two potential targets for innovative therapeutic approaches [27].

As previously reported, some of the manifestations seen in sarcopenia have been observed in mice exposed to cadmium, suggesting that heavy metal intoxication may contribute to the progressive loss of muscle mass that occurs during aging [4]. Therefore, we analyze below the histological and immunohistochemical profile of muscle tissue from a patient with metallosis-induced cobalt–chromium hip replacement, investigating the presence of some important molecular signs of muscle degeneration, such as increased expression of myostatin and NOX4.

## 2. Case Report Description

**Figure 1:**
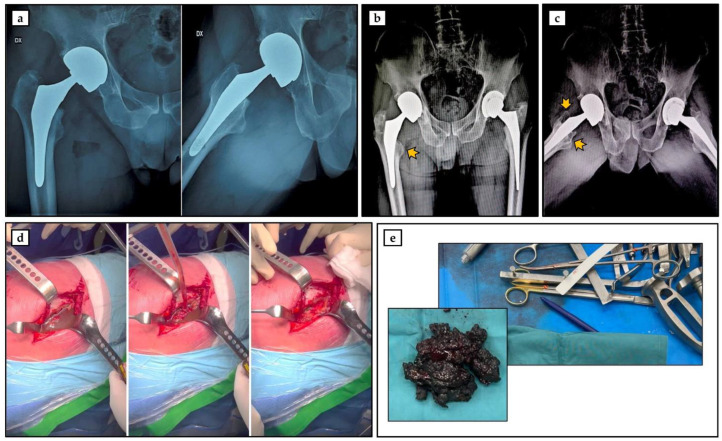
Instrumental and surgical evaluation of the patient. (**a**) Radiographic image of Mitch trh V40 joint prosthesis with cobalt–chrome alloy interface implanted in the right hip in March 2008. (**b**) Radiographic image of June 2022 taken following left total hip replacement surgery. The arrow shows discrete bone loss around the right hip prosthesis. (**c**) Radiographic image from June 2023 showing further acetabular bone loss around the right hip prosthesis (arrows). (**d**,**e**) Intraoperative image showing abundant intra-articular fluid and metallosic degeneration of periarticular tissues.

A 75-year-old Caucasian man came to our attention at the Department of Orthopaedics and Traumatology of the Policlinico “Tor Vergata” in June 2022 for left coxarthrosis. The clinical and anamnestic investigation showed that the patient, a former professional rugby player, was in good health, as he was a non-smoker and had a varied and balanced diet. In March 2008, the patient underwent hip arthroplasty surgery for severe right coxarthrosis, during which a Mitch trh V40 joint replacement with a cobalt–chromium alloy interface was implanted (Figure 1a). Following the left total hip replacement surgery performed at our hospital, serum and urine assays to determine chromium and cobalt metal ion levels were suggested to the patient, considering the mechanical loosening of the prosthesis associated with a large area of bone resorption (Figure 1b). Not surprisingly, tests performed by the patient in January 2023 reported elevated serum and urinary levels of chromium and cobalt ions (Table 1), highlighting the need to replace the prosthetic implant with a chromium–cobalt interface.

During the pre-hospitalization phase in June 2023, endocrine, neoplastic, and neurodegenerative diseases were ruled out, along with drug and metal allergies. Radiographic evaluation revealed additional acetabular tissue loss, which was responsible for the previously observed mechanical loosening (Figure 1c). Furthermore, clinical and instrumental investigations showed *T*-score values (L1–L4, femoral neck, and total femur) of −0.9, −0.1, and 1.3, respectively, while the serum levels of parathormone (PTH) and 25-(OH)-VitD were 55.0 pg/mL and 15.4 ng/mL, respectively. Of note, the patient was completely asymptomatic, as he did not report the presence of joint pain and had excellent functional capacity, with a Harris hip score (HHS) of 100.

In the first week of July 2023, the patient underwent revision surgery for a right hip replacement, during which the presence of abundant intra-articular fluid was noted, which was aspirated, in association with metallosic degeneration of the periarticular tissues, which were excised (Figure 1d,e). At this stage, a sample of muscle tissue was taken for biological analysis.

## 3. Materials and Methods

### 3.1. Participants

During hip replacement revision surgery, biopsies of the vastus lateralis muscle were taken and subsequently processed for qualitative and quantitative investigations. All experimental procedures were performed according to the World Medical Association’s Code of Ethics (Declaration of Helsinki) and were conducted with the approval of the Ethics Committee of the Policlinico “Tor Vergata” (approval reference number #17/21).

In our study, a patient of equal age and sex who underwent the same hip arthroplasty for coxarthrosis and the same clinical and experimental procedures was included as a control. His clinical characteristics are shown in Supplementary Table S1. Informed consent was obtained from each patient prior to surgery.

### 3.2. Histological and Morphometric Analysis

Muscle biopsies were collected for histological and morphometric analysis. The samples were immediately fixed in 4% paraformaldehyde for 24 h and embedded in paraffin. The 3 μm-thick sections were stained with hematoxylin and eosin (H&E) (Bio-Optica, Milan, Italy), and the slides were visualized by a Nikon upright microscope ECLIPSE Ci-S (Nikon Corporation, Tokyo, Japan) connected to a Nikon digital camera. Images were acquired at 20× magnification using NIS-Elements software (5.30.01; Laboratory Imaging, Prague, Czech Republic).

Morphometric analysis was conducted by two blinded observers who assessed the cross-sectional area of the muscle fibers and the fat infiltration area between the fibers. Measurements were performed at 40× magnification, taking a total of 8 non-overlapping readings for each patient. In addition, the reference area used for the morphometric analysis was set up using the NIS-Elements software so that the size of the region of interest was the same at each evaluation.

### 3.3. Immunohistochemistry

An immunohistochemical analysis was conducted to assess myostatin and NADPH Oxidase 4 (NOX4) expressions in muscle tissue. The 3 μm-thick sections were pre-treated with EDTA citrate (pH 6.0) for 20 min at 95 °C and then incubated for 1 h with rabbit polyclonal anti-myostatin propeptide (clone ab134682, AbCam, Cambridge, UK) or rabbit polyclonal anti-NOX4 (NB110–58849, Novus Biologicals, Centennial, CO, USA). Washings were performed with phosphate-buffered saline (PBS)/Tween20 (pH 7.6) (UCS Diagnostic, Rome, Italy); the horseradish peroxidase (HRP)-3,3′ diaminobenzidine (DAB) detection kit (UCS Diagnostic, Rome, Italy) was used to reveal immunohistochemical reactions. Specifically, 50 μL of DAB/450 μL of substrate were incubated for 3 min. The immunostaining background was evaluated with negative controls for each reaction (Supplementary Figure S1) obtained by incubating the sections with secondary antibodies only (HRP) or with the detection system only (DAB).

Immunopositive cells for myostatin and NOX4 were detected using NIS-Elements software (5.30.01; Laboratory Imaging, Prague, Czech Republic) and expressed as a percentage of the total analyzed for myostatin and NOX4. For each condition, the experiment was conducted in triplicate (*n* = 15 from *N* = 5 experiments).

### 3.4. Statistical Analysis

Statistical analysis was performed using GraphPad Prism 8 software (GraphPad Prism 8.0.1, La Jolla, CA, USA). All data were expressed as mean ± standard error and were compared by unpaired t-test with Welch’s correction. The data were considered significantly different if *p* < 0.05.

## 4. Results

**Figure 2:**
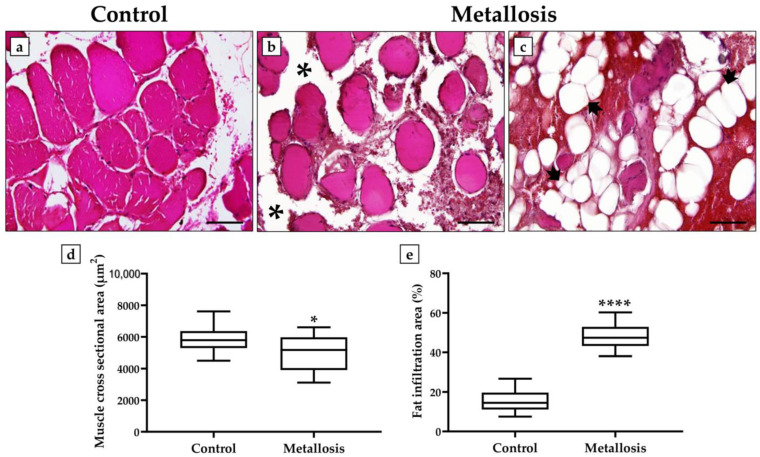
Hematoxylin and eosin (H&E)-stained sections of muscle tissue from control and metallosis patients. (**a**) The muscle tissue of the control patient was characterized by well-organized muscle fascicles with polygonal and multinucleated fibers and peripheral nuclei. (**b**) The muscle tissue of the metallosis patient was markedly degenerated, with isolated muscle fibers and abundant adipose tissue (asterisks). (**c**) Numerous adipocytes (arrows) densely interposed between muscle fibers were detected in metallosis patients. (**d**) The cross-sectional area of muscle fibers is shown for each patient (* *p* < 0.05). (**e**) The fat infiltration area in muscle is shown for each patient (**** *p* < 0.0001). For 20× images, scale bar represents 100 μm.

Histological analysis of the control patient’s muscle tissue showed normal tissue architecture, with polygonal and multinucleated skeletal muscle fibers with peripheral nuclei in association with a clear organization of the muscle fibers into fascicles (Figure 2a). In contrast, extensive heterogeneity was evidenced in the muscle tissue of the metallosis patient. In fact, some areas were characterized by poorly organized muscle fibers, generally isolated and surrounded by abundant adipose tissue (Figure 2b), while other areas showed a true accumulation of adipose tissue, with numerous adipocytes densely interposed between them and almost absent muscle fibers (Figure 2c).

The morphometric data confirmed the histological analysis, as demonstrated by the significant reduction in the cross-sectional area of the muscle fibers and a significant increase in fat infiltration area in the presence of metallosis. In fact, the values of the cross-sectional area of the muscle fibers were 5892.9 ± 249.2 in the control patient and 5043.5 ± 295.5 in the metallosis patient (* *p* < 0.05) (Figure 2d), while the values of the fat infiltration area expressed as a percentage of the total area analyzed were 15.7 ± 1.4 in the control patient and 48.7 ± 1.8 in the metallosis patient (**** *p* < 0.0001) (Figure 2e).

An immunohistochemical analysis was performed to investigate a possible association between the metallosis condition and changes in the expression of myostatin, the main negative regulator of muscle growth, and NOX4, an indicator of oxidative stress. Cells positive for myostatin and NOX4 were expressed as a percentage of the total analyzed.

**Figure 3:**
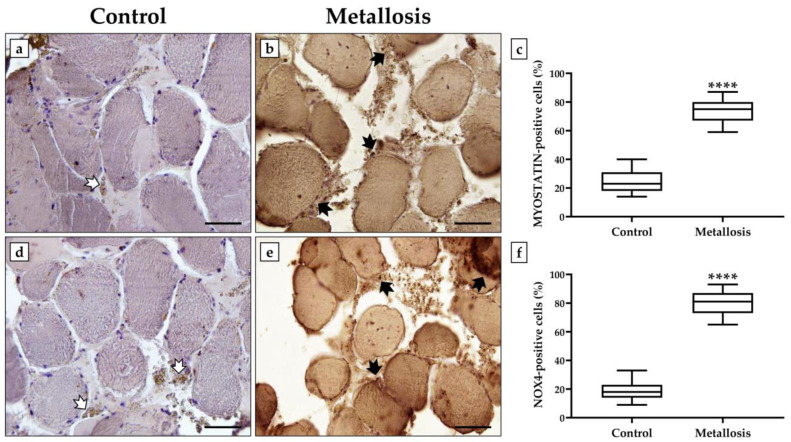
Myostatin and NADPH Oxidase 4 (NOX4) expression analysis in muscle tissue of control and metallosis patients. (**a**–**c**) The highest levels of myostatin expression (black arrows) were detected in the muscle tissue of the metallosis patient compared to the control patient, where the number of myostatin-positive cells was significantly reduced (white arrow). (**** *p* < 0.0001). (**d**–**f**) The metallosis condition significantly increases oxidative stress detected in the muscle tissue by NOX4-positive cell counts (black arrows) compared to the control patient characterized by a significant reduction in NOX4 expression (white arrows) (**** *p* < 0.0001). For 20× images, scale bar represents 100 μm.

Figure 3 shows that the expression levels of myostatin and NOX4 are markedly elevated in the presence of metallosis. Indeed, the relative number of cells positive for myostatin was 24.9 ± 1.9 in the control patient and 73.7 ± 2.2 in the metallosis patient (**** *p* < 0.0001) (Figure 3a–c). Similarly, the relative number of positive cells for NOX4 was 19.0 ± 1.8 in the control patient and 80.3 ± 2.2 in the metallosis patient (**** *p* < 0.0001) (Figure 3d–f).

## 5. Discussion

Chronic exposure to heavy metals has serious consequences both locally and systemically [28]. Indeed, in addition to dramatically damaging the musculoskeletal system, such substances can, in severe cases, impair vision and hearing, lead to weight and strength loss, and promote the development of skin diseases and neoplastic growth [29,30,31]. Unfortunately, the metallosis consequences on human skeletal muscle are poorly characterized, highlighting the need for cohort studies to investigate this phenomenon. Therefore, the aim of our study was to examine muscle changes in a patient undergoing revision hip replacement with elevated blood and urinary concentrations of chromium and cobalt. Importantly, the elevated concentration of these heavy metals did not lead to the appearance of neuroendocrine symptoms or the development of neoplastic formations. However, histological and immunohistochemical analyses revealed that the enrolled patient’s vastus lateralis muscle showed dramatic structural alterations and a massive infiltration of adipose tissue between the muscle fibers, as well as alterations in the expression pattern of important biomarkers of musculoskeletal health and oxidative stress, such as myostatin and NOX4, all hallmarks of sarcopenia [32].

Particularly, myostatin is the main negative regulator of muscle growth, as its expression is up-regulated under unloaded and sedentary conditions and down-regulated under conditions of muscle hypertrophy [33,34]. Of note, high myostatin expression could compromise not only the quality of muscle tissue but also the quality of the entire musculoskeletal system. Indeed, given the mutual interaction between muscle and bone tissue, a condition of muscle atrophy due to increased levels of myostatin could favor bone mass loss due to the reduced load forces applied by muscles to bone [35,36]. Furthermore, Qin et al. described an anti-osteogenic role of myostatin by observing an over-production of negative regulators of bone growth by osteocytes in response to this myokine [37].

Importantly, muscle tissue and adipose tissue share the same progenitor, mesenchymal stem cells. Myostatin, known for its ability to promote adipogenesis, could play a key role in determining cell fate towards a myogenic or adipogenic pathway, acting as a regulator controlling body fat mass [17,38]. Such alterations in the musculoskeletal system inevitably occur during aging, predisposing to age-related bone and muscle diseases. The chronic presence of metal debris could lead to a condition of oxidative stress in the muscle and promote myostatin production, favoring adipose infiltration between muscle fibers. Fortunately, cases of metallosis due to prosthetic implants with a metal–metal interface are increasingly rare, mainly due to the introduction of biocompatible and very low-impact materials in the medical field [39]. However, Sahan et al. published the results of a systematic literature review analyzing cases of metallosis after total knee arthroplasty (TKA) surgery in 2020. The authors collected 29 studies on cases of metallosis after TKA, highlighting the need to investigate the impact, in molecular terms, of heavy metals on the musculoskeletal system [40].

## 6. Conclusions

Although metallosis is a rare complication of arthroplasty surgeries, the potential risks of chronic heavy metal infiltration into musculoskeletal tissues and the very serious impact on patient health should be considered. Importantly, exercise is a well-known powerful strategy to preserve musculoskeletal health by reducing adipose infiltration and increasing muscle mass. Therefore, we hypothesize that the absence of a marked condition of muscle atrophy, typical of a geriatric patient and normally aggravated in the case of metallosis, is strictly dependent on the intense physical activity practiced by the patient in previous years. However, further studies are needed to investigate the molecular alterations in metallosis patients and to intervene at an early stage with a systemic diagnostic approach that allows the cause of metallosis to be identified and eliminated as part of the revision surgery.

## Figures and Tables

**Table 1:** Clinical characteristics of metallosis patient.

Parameters	Values
Age (years)	75
BMI (Kg/cm^2^)	24.7
*T*-score (L1–L4)	−0.9
*T*-score (femoral neck)	−0.1
*T*-score (total femur)	1.3
PTH (pg/mL)	55.0
25-(OH)-VitD (ng/mL)	15.4
HHS	100
Cr (μg/L)	2.7
Co (μg/L)	2.1
Urinary Cr (mcg/g)	4.7
Urinary Co (mcg/L)	14.7

BMI: bone mass index; PTH: parathormone; HHS: Harris hip score; Cr: chrome (reference value: less than 1 in exposed subjects); Co: cobalt (reference value: less than 1 in exposed subjects); Urinary Cr: urinary chrome (reference value: less than 25.0 in exposed subjects; less than 2.0 in unexposed subjects); Urinary Co: urinary cobalt (reference value: less than 15.0 in exposed subjects; 0.2–2.0 in unexposed subjects).

## Data Availability

The data presented in this study are available upon request from the corresponding author.

## Supplementary Materials

The following supporting information can be downloaded at https://www.mdpi.com/article/10.3390/geriatrics8050092/s1. Table S1: Clinical characteristic of the control patient; Figure S1: Immunohistochemical analysis of muscle tissue.

## References

[1] Banjabi A.A., Kurunthachalam K., Kumosani T.A., Abulnaja K.O., Al-Malki A.L., Moselhy S.S. (2022). Serum heavy metals of passive smoker females and its correlation to bone biomarkers and risk of osteoporosis. Environ. Sci. Pollut. Res. Int..

[2] Ximenez J.P.B., Zamarioli A., Kacena M.A., Barbosa R.M., Barbosa F.J. (2021). Association of Urinary and Blood Concentrations of Heavy Metals with Measures of Bone Mineral Density Loss: A Data Mining Approach with the Results from the National Health and Nutrition Examination Survey. Biol. Trace Elem. Res..

[3] Scimeca M., Feola M., Romano L., Rao C., Gasbarra E., Bonanno E., Brandi M.L., Tarantino U. (2017). Heavy metals accumulation affects bone microarchitecture in osteoporotic patients. Environ. Toxicol..

[4] He H., Lin X., Tong T., Xu Y., Hong H., Zhang J., Xu Y., Huang C., Zhou Z. (2023). Cadmium exposure impairs skeletal muscle function by altering lipid signature and inducing inflammation in C57BL/6J mice. Ecotoxicol. Environ. Saf..

[5] Rony L., Lancigu R., Hubert L. (2018). Intraosseous metal implants in orthopedics: A review. Morphologie.

[6] Alm J.J., Moritz N., Aro H.T. (2016). In vitro osteogenic capacity of bone marrow MSCs from postmenopausal women reflect the osseointegration of their cementless hip stems. Bone Rep..

[7] Gasbarra E., Perrone F.L., Celi M., Rao C., Feola M., Cuozzo N., Tarantino U. (2013). Total hip arthroplasty revision in elderly patients. Aging Clin. Exp. Res..

[8] Ikeda T., Takahashi K., Kabata T., Sakagoshi D., Tomita K., Yamada M. (2010). Polyneuropathy caused by cobalt-chromium metallosis after total hip replacement. Muscle Nerve.

[9] Czekaj J., Ehlinger M., Rahme M., Bonnomet F. (2016). Metallosis and cobalt-chrome intoxication after hip resurfacing arthroplasty. J. Orthop. Sci. Off. J. Jpn. Orthop. Assoc..

[10] Marzetti E. (2022). Musculoskeletal Aging and Sarcopenia in the Elderly. Int. J. Mol. Sci..

[11] Bonanni R., Gino Grillo S., Cariati I., Tranquillo L., Iundusi R., Gasbarra E., Tancredi V., Tarantino U. (2023). Osteosarcopenia and Pain: Do We Have a Way Out?. Biomedicines.

[12] Cruz-Jentoft A.J., Bahat G., Bauer J., Boirie Y., Bruyère O., Cederholm T., Cooper C., Landi F., Rolland Y., Sayer A.A. (2019). Sarcopenia: Revised European consensus on definition and diagnosis. Age Ageing.

[13] Jo D., Yoon G., Kim O.Y., Song J. (2022). A new paradigm in sarcopenia: Cognitive impairment caused by imbalanced myokine secretion and vascular dysfunction. Biomed. Pharmacother..

[14] Dao T., Green A.E., Kim Y.A., Bae S.-J., Ha K.-T., Gariani K., Lee M.-R., Menzies K.J., Ryu D. (2020). Sarcopenia and Muscle Aging: A Brief Overview. Endocrinol. Metab..

[15] Bilski J., Pierzchalski P., Szczepanik M., Bonior J., Zoladz J.A. (2022). Multifactorial Mechanism of Sarcopenia and Sarcopenic Obesity. Role of Physical Exercise, Microbiota and Myokines. Cells.

[16] Scimeca M., Piccirilli E., Mastrangeli F., Rao C., Feola M., Orlandi A., Gasbarra E., Bonanno E., Tarantino U. (2017). Bone Morphogenetic Proteins and myostatin pathways: Key mediator of human sarcopenia. J. Transl. Med..

[17] Deng B., Zhang F., Wen J., Ye S., Wang L., Yang Y., Gong P., Jiang S. (2017). The function of myostatin in the regulation of fat mass in mammals. Nutr. Metab..

[18] Guo T., Jou W., Chanturiya T., Portas J., Gavrilova O., McPherron A.C. (2009). Myostatin inhibition in muscle, but not adipose tissue, decreases fat mass and improves insulin sensitivity. PLoS ONE.

[19] Konopka A.R., Wolff C.A., Suer M.K., Harber M.P. (2018). Relationship between intermuscular adipose tissue infiltration and myostatin before and after aerobic exercise training. Am. J. Physiol. Regul. Integr. Comp. Physiol..

[20] Sullivan-Gunn M.J., Lewandowski P.A. (2013). Elevated hydrogen peroxide and decreased catalase and glutathione peroxidase protection are associated with aging sarcopenia. BMC Geriatr..

[21] Lian D., Chen M.-M., Wu H., Deng S., Hu X. (2022). The Role of Oxidative Stress in Skeletal Muscle Myogenesis and Muscle Disease. Antioxidants.

[22] Wu S.-E., Hsu J.-C., Chang Y.-L., Chuang H.-C., Chiu Y.-L., Chen W.-L. (2022). Benzo[a]pyrene exposure in muscle triggers sarcopenia through aryl hydrocarbon receptor-mediated reactive oxygen species production. Ecotoxicol. Environ. Saf..

[23] Marzetti E., Calvani R., Cesari M., Buford T.W., Lorenzi M., Behnke B.J., Leeuwenburgh C. (2013). Mitochondrial dysfunction and sarcopenia of aging: From signaling pathways to clinical trials. Int. J. Biochem. Cell Biol..

[24] Alway S.E., Siu P.M. (2008). Nuclear apoptosis contributes to sarcopenia. Exerc. Sport. Sci. Rev..

[25] Huo F., Liu Q., Liu H. (2022). Contribution of muscle satellite cells to sarcopenia. Front. Physiol..

[26] Antuña E., Cachán-Vega C., Bermejo-Millo J.C., Potes Y., Caballero B., Vega-Naredo I., Coto-Montes A., Garcia-Gonzalez C. (2022). Inflammaging: Implications in Sarcopenia. Int. J. Mol. Sci..

[27] Tu H., Li Y.-L. (2023). Inflammation balance in skeletal muscle damage and repair. Front. Immunol..

[28] Oliveira C.A., Candelária I.S., Oliveira P.B., Figueiredo A., Caseiro-Alves F. (2015). Metallosis: A diagnosis not only in patients with metal-on-metal prostheses. Eur. J. Radiol. Open.

[29] Wolfson M., Curtin P., Curry E.J., Cerda S., Li X. (2020). Giant cell tumor formation due to metallosis after open latarjet and partial shoulder resurfacing. Orthop. Rev..

[30] Green B., Griffiths E., Almond S. (2017). Neuropsychiatric symptoms following metal-on-metal implant failure with cobalt and chromium toxicity. BMC Psychiatry.

[31] Jayasekera N., Gouk C., Patel A., Eyres K. (2015). Apparent Skin Discoloration about the Knee Joint: A Rare Sequela of Metallosis after Total Knee Replacement. Case Rep. Orthop..

[32] Cariati I., Bonanni R., Onorato F., Mastrogregori A., Rossi D., Iundusi R., Gasbarra E., Tancredi V., Tarantino U. (2021). Role of Physical Activity in Bone-Muscle Crosstalk: Biological Aspects and Clinical Implications. J. Funct. Morphol. Kinesiol..

[33] Cariati I., Scimeca M., Bonanni R., Triolo R., Naldi V., Toro G., Marini M., Tancredi V., Iundusi R., Gasbarra E. (2022). Role of Myostatin in Muscle Degeneration by Random Positioning Machine Exposure: An in vitro Study for the Treatment of Sarcopenia. Front. Physiol..

[34] Elkasrawy M.N., Hamrick M.W. (2010). Myostatin (GDF-8) as a key factor linking muscle mass and bone structure. J. Musculoskelet. Neuronal Interact..

[35] Bonanni R., Cariati I., Marini M., Tarantino U., Tancredi V. (2023). Microgravity and Musculoskeletal Health: What Strategies Should Be Used for a Great Challenge?. Life.

[36] Palmieri M., Cariati I., Scimeca M., Pallone G., Bonanno E., Tancredi V., D’Arcangelo G., Frank C. (2019). Effects of short-term aerobic exercise in a mouse model of Niemann-Pick type C disease on synaptic and muscle plasticity. Ann. Ist. Super. Sanita.

[37] Qin Y., Peng Y., Zhao W., Pan J., Ksiezak-Reding H., Cardozo C., Wu Y., Divieti Pajevic P., Bonewald L.F., Bauman W.A. (2017). Myostatin inhibits osteoblastic differentiation by suppressing osteocyte-derived exosomal microRNA-218: A novel mechanism in muscle-bone communication. J. Biol. Chem..

[38] Tang L., Yang X., Gao X., Du H., Han Y., Zhang D., Wang Z., Sun L. (2016). Inhibiting myostatin signaling prevents femoral trabecular bone loss and microarchitecture deterioration in diet-induced obese rats. Exp. Biol. Med..

[39] Piccirilli E., Cariati I., Primavera M., Triolo R., Gasbarra E., Tarantino U. (2022). Augmentation in fragility fractures, bone of contention: A systematic review. BMC Musculoskelet. Disord..

[40] Sahan I., Anagnostakos K. (2020). Metallosis after knee replacement: A review. Arch. Orthop. Trauma. Surg..

