# Supplementary material for: Metallosis after Hip Arthroplasty Damages Skeletal Muscle: A Case Report
Source: Geriatrics (Basel). 2023 Sep 15;8(5):92. doi: 10.3390/geriatrics8050092 (PMC10514854; doi:10.3390/geriatrics8050092)
Supplement: Supplementary file 1 [file geriatrics-08-00092-s001.zip › geriatrics-2578877-supplementary.pdf]

Table S1. Clinical characteristics of the control patient.

Parameters	Values
Age (years)	75
BMI (Kg/cm^2)	25.8
T-score (L1–L4)	-1.1
T-score (femoral neck)	-0.2
T-score (total femur)	1.2
PTH (pg/mL)	59.2
25-(OH)-VitD (ng/mL)	18.7
HHS	85

BMI: bone mass index; PTH: parathormone; HHS: harris hip score.

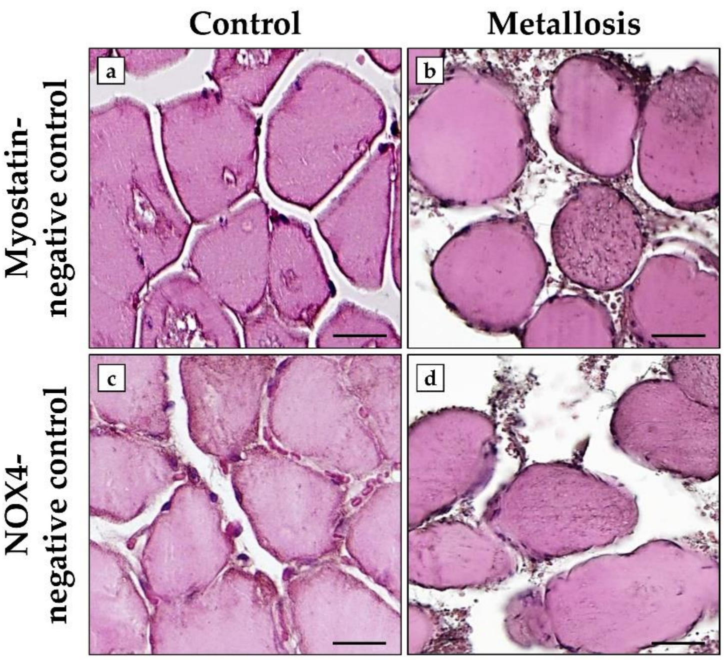

Figure S1. Immunohistochemical analysis for myostatin and NADPH Oxidase 4 (NOX4) expressions in muscle tissue. (a) Myostatin-negative control in the control patient. (b) Myostatin-negative control in the metallosis patient. (c) NOX4-negative control in the control patient. (d) NOX4-negative control in the metallosis patient. Images were magnified 20×, scale bar represents 100 μm.
